# Novel approaches for the rational design of PROTAC linkers

**DOI:** 10.37349/etat.2020.00023

**Published:** 2020-10-30

**Authors:** Almaz Zagidullin, Vasili Milyukov, Albert Rizvanov, Emil Bulatov

**Affiliations:** 1 Institute of Fundamental Medicine and Biology, Kazan Federal University, 420008 Kazan, Russia; 2 A.E. Arbuzov Institute of Organic and Physical Chemistry, FRC Kazan Scientific Center of RAS, 420088 Kazan, Russia; 3 Shemyakin-Ovchinnikov Institute of Bioorganic Chemistry, Russian Academy of Sciences, 117997 Moscow, Russia; University of Southampton, UK

**Keywords:** PROTAC, protein degradation, linker chemistry

## Abstract

Proteolysis targeting chimeras (PROTACs) represent a promising class of hetero-bivalent molecules that facilitate ubiquitination of a target protein by simultaneously binding and bringing together both the E3 enzyme and the target. These compounds consist of three structural components: two ligands one of which binds the protein of interest (POI) while the other binds an E3 ubiquitin ligase to promote POI ubiquitination, and a linker connecting both moieties. Recent developments in the field highlight the fact that linker composition and length play a crucial role in achieving optimal PROTAC properties, modulate binding kinetics and substantially impacts the potency and selectivity. In this review, the authors briefly discuss the recent findings in PROTAC design approaches with focus on the linker. For each PROTAC such linker parameters as chemical nature, length, hydrophilicity and rigidity have to be optimized to achieve improved stability, bioavailability cell membrane permeability and suitable spatial orientation between the target POI and the E3 ubiquitin ligase. Thus rational linker design with respect to composition, length and attachment sites is essential for the development of potent PROTAC compounds. Computer-aided design and novel innovative linker strategies, such as PROTAC shortening, photo-switchable PROTACs, in-cell click-formed CLIPTACs, “click chemistry” approaches are also discussed in the review.

## Introduction

**Figure 1.**
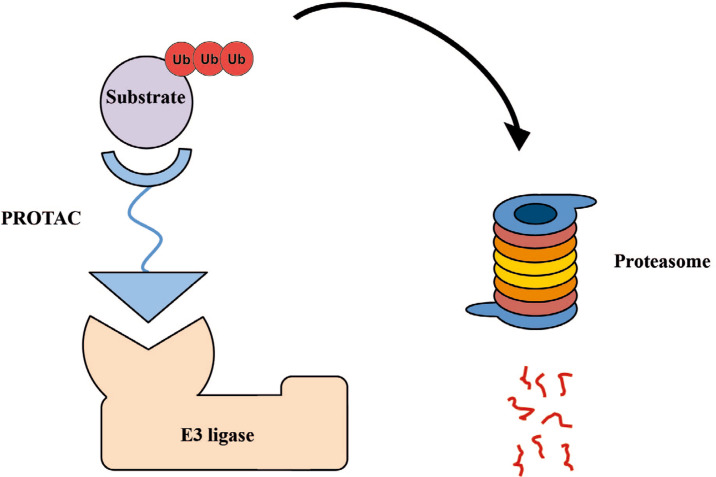
Schematic representation of PROTAC molecule acting to induce ubiquitination and subsequent proteasomal degradation of substrate POI (image adapted from [1] and modified). These small molecule degraders contain substrate-specific component, short linker, and E3-specific component

Proteolysis targeting chimeras (PROTACs), also known as degraders, degronimids, protein domain parser (PDPs), specific and non-genetic IAP-dependent protein erasers (SNIPERs), represent a promising class of hetero-bivalent molecules that promote ubiquitination and subsequent proteasomal degradation of the target protein. PROTACs consist of three structural components that allow bringing together the E3 ubiquitin ligase and the target: a ligand targeting the protein of interest (POI), a second ligand binding an E3 ubiquitin ligase [1] to promote POI ubiquitination, and a linker connecting both moieties. This approach, sometimes called as “chemical knockdown”, enables ligand-induced degradation of specific endogenous proteins [2]. The main advantage of PROTACs is their catalytic nature that enables sub-stoichiometric and catalytic enzymatic removal of substrate POIs rather than their inhibition via occupation of the binding site (Figure 1). This strategy has been extensively used in degrading a variety of therapeutically relevant proteins, transcriptional regulators and hormone receptors.

In general, PROTACs are designed as small molecules, since stability, bioavailability and cell membrane permeability depend on properties and molecular weight of the both ligands and the linker. For each PROTAC compound such parameters of the linker as chemical nature, length, hydrophilicity and rigidity have to be optimized to achieve improved cell permeability and suitable spatial orientation between the target POI and the E3 ubiquitin ligase [3, 4].

In a recent study, conformation of bromodomain-containing protein (BRD)4/cereblon (CRBN) protein complex was found to be linker-dependent suggesting that PROTACs with the same E3-recruiting and POI-recruiting ligands might have different selectivity profiles depending on chemical composition and linker attachment points [5]. Furthermore, linker length also affects the degradation profile: an increment of just 3 atoms in lapatinib-based PROTAC converted the dual epidermal growth factor receptor (EGFR) and human epidermal growth factor receptor 2 (HER2) selectivity to EGFR only [6]. Linker length and composition have a profound effect on PROTAC properties, e.g., HaloPROTACs without ethylene glycol do not induce degradation of their targets [7]; B-cell receptor (BCR)-tyrosine-protein kinase (ABL) PROTACs with an ether instead of an amide demonstrate higher cell permeability [8]. In addition, methyl bestatin based PROTACs can function as dual cellular retinoic acid-binding protein (CRABP)-I/II degraders, whereby the longer polyethylene glycol (PEG)-linker shifts the degradation selectivity towards CRABP-I and the shorter one–towards CRABP-II [9].

In the last decade, extensive efforts have been made to improve PROTAC design, especially concerning formation of heteromeric protein complexes and optimization of linker properties as the main driving factors for effective POI degradation. Recent developments in the field highlight the fact that linker composition and length play a crucially important role in achieving optimal physicochemical PROTAC properties and effective recruitment of POI. In this review, we discuss the recent findings in PROTAC design with primary focus on the linker.

## Linker classification

The role of the linker is often underestimated, yet it exerts a defining effect on physicochemical properties and biological activity of PROTAC compounds. The right combination of length, hydrophilicity and rigidity of ligand-connecting linkers form a basis for successful design of highly potent PROTACs. A wide variety of linkers are most commonly based on PEGs, unsaturated alkane chains and triazoles (Table 1). Linkers are typically attached to the ligands through amines, amides, ethers, alkylamines, single and multiple C-C bonds. Some linkers have a certain degree of flexibility, e.g., PEGs or (un)saturated alkanes, while the others are relatively rigid, e.g., C-C multiple bonds, piperidines and triazoles. A piperidine group was successfully used as linker in order to increase the solubility of androgen receptor (AR) degrader PROTACs [10]. Most linkers contain a combination of hydrophobic (unsaturated alkanes) and hydrophilic moieties (PEGs, piperidines, amides, triazoles, etc.) to balance log *P* of the resulting compounds [11, 12].

**Table 1.** The common types of linkers used for the design of PROTACs

**Flexible linkers (n, m = 0, 1, 2, 3, 4, etc.)**	**Relatively rigid linkers**	**Click chemistry based linkers**
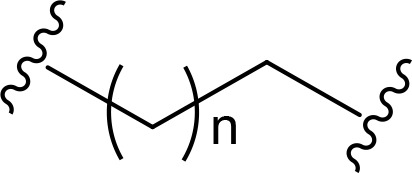 **alkane**	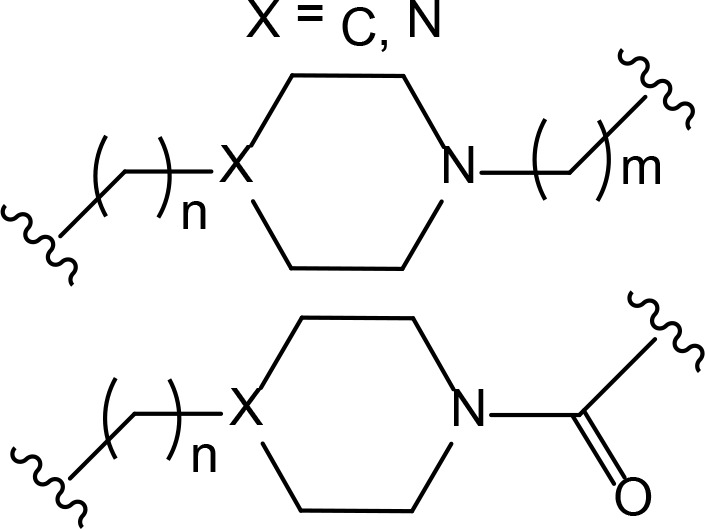 **piperidine/benzene**	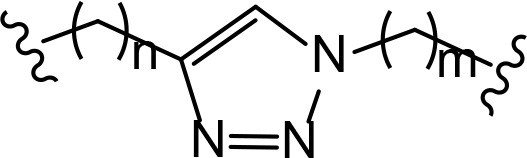 **triazole**
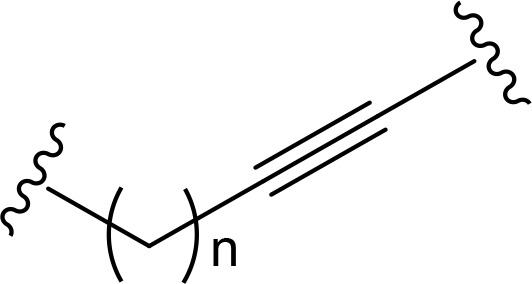 **alkyne-alkane**		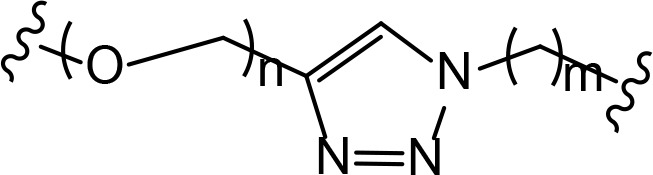 **triazole-PEG**
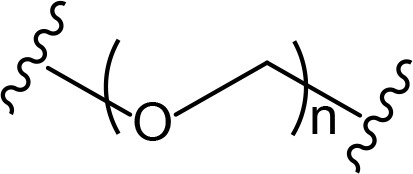 **PEG**	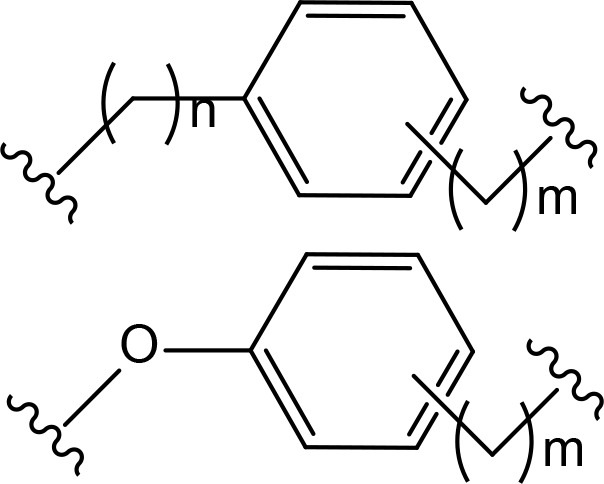 **benzene**	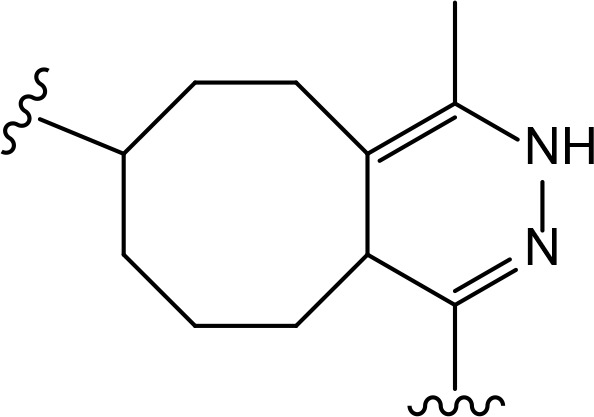 **(CLIPTAC)**
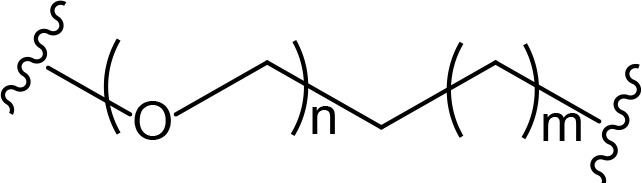 **PEG-alkane**		
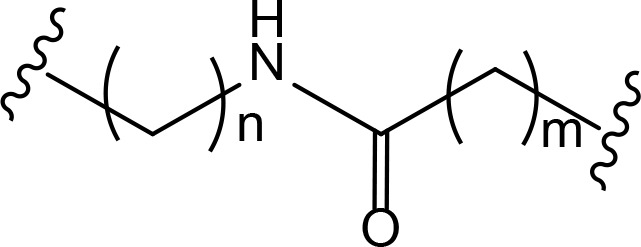 **amide**	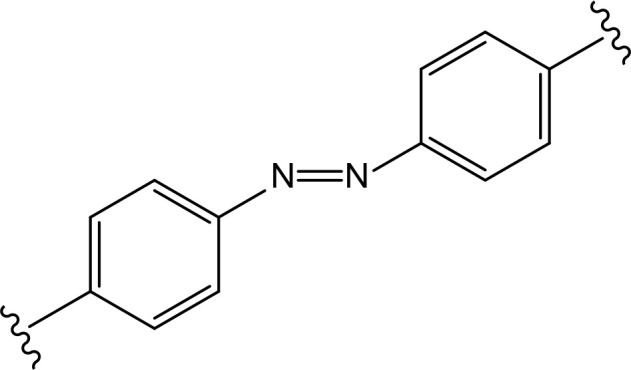 **azobenzene (photoswitchable)**	

### Alkyl- and PEG-based linkers

One of the first PROTAC prototypes was reported in as early as 2001 by Sakamoto et al. [13], and contained ovalicin bound to methionine aminopeptidase 2 (MetAP-2), an aliphatic linker and an inhibitor of nuclear factor kappa-B alpha (IκBα) phospho-peptide recognized by SCF (Skp, Cullin, F-box) ubiquitin ligase. Currently, flexible and cheap linear saturated [14–17] and linear unsaturated aliphatic linkers with alkenyl [18] and alkynyl chains [19–21] are most commonly used for PROTAC design.

PEG linkers are essential and privileged scaffold elements for PROTACs design and synthesis. Soluble and flexible PEG linkers provide significant contribution to protein-ligand interaction within the ternary complex and stabilize its orientation by cooperative binding [22]. For example, PEG linkers provide crucial van der Waals interactions with the αB and αC (BC loop) of BRD4^BD2^ and also the hydrogen bond between ether oxygen adjacent to JQ1 amide linkage and bromodomain (BD)2-specific His437 [23].

At the same time linkers with aliphatic and/or ether chains are often associated with increased risk of oxidative metabolism that depends on the accessibility to catalytic sites of the oxidizing enzymes.

### Triazole and “click chemistry”-based linkers

“Click chemistry” reactions provide an accessible and tunable strategy for the selection of synthetic linkers and lay a solid foundation for the development of a diverse library of PROTACs. Currently, the latest applications of “click chemistry” are based on Cu-catalyzed azide-alkyne cycloaddition and the Diels-Alder reaction. The copper-catalyzed “click” reaction between azides and alkynes allows parallel synthesis of PROTACs libraries with triazole linkers [24–26]. Recently, Wurz et al. [27], performed the first comprehensive study on the relationship between linker length and activity for PROTACs generated by means of “click” reaction.

**Figure 2.**
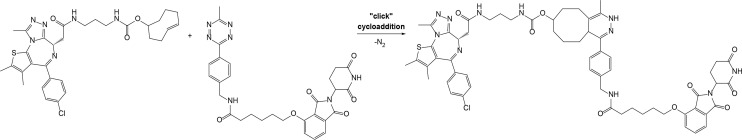
Representative scheme explaining the self-assembly of in-cell click-formed PROTACs (CLIPTACs)

“Click chemistry” approach was successfully used by Astex Therapeutics in Cambridge to discover first in-cell click-formed proteolysis targeting chimeras (CLIPTACs), a variation of PROTACs, with two precursor components combined by bio-orthogonal “click” reaction [28]. The resulting CLIPTAC compound successfully degraded two primary targets in cancer–BRD4 and extracellular signal-regulated kinases (ERK)1/2. Authors designed a tetrazine-labeled thalidomide derivative that reacts rapidly with the *trans*-cyclooctene-labeled ligand of the target protein to form a CRBN-based PROTAC (Figure 2). Intracellular self-assembly of CLIPTACs may help to overcome the molecular weight limits, improve drug-like properties, solubility and cell permeability of the compounds. Altogether this will expand the inhibitor toolkit available to biologists and facilitate uncovering the disease-associated molecular mechanisms.

In addition to PEGs, other popular linker components include triazole moieties that were also identified as privileged scaffolds for the development of PROTACs [29]. In case of Wogonin-based PROTACs selective for cyclin-dependent kinase 9 (CDK9), a triazole linker resulted in higher efficiency than an alkane chain [30]. Overall, “click chemistry” approach represents a powerful tool to generate novel PROTAC libraries as it can be easily applied to a wide range of E3 ligases and target proteins.

### Innovative linkers

In recent years, a lot of attention has been drawn to the development of innovative PROTAC linker technologies. By incorporating the azobenzene photo-switch into the linker region of dBET1 or dFKBP-1, a light-inducible PROTAC (also named PHOTACs) has been obtained that can be switched on and off by 390 nm and 525 nm wavelengths, respectively [31]. Pfaff et al. [32], have developed similar azobenzene photo-switchable PROTACs (photoPROTACs) for Von Hippel-Lindau protein (VHL)-mediated degradation of BRD proteins. In this case, *ortho*-F4-azobenzene structurally incorporated into the linker could exist in two interconvertible conformations–inactive azo-*cis*-isomer and active azo-*trans*-isomer. The photoPROTAC gets activated by 415 nm and inactivated by 530 nm light source.

**Figure 3.**
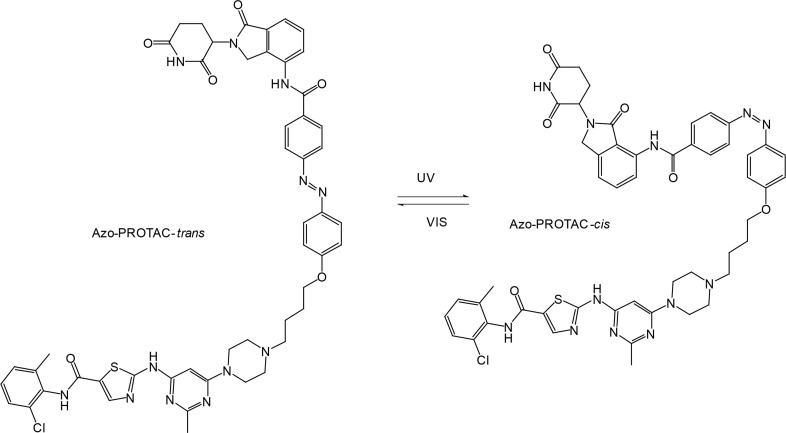
Azo-PROTACs: novel light-controlled PROTACs

Another interesting case includes lenalidomide-azobenzene-dasatinib trifunctional system (also named Azo-PROTAC) that demonstrated marked differences in the abilities of *trans*- and *cis*-isomers of Azo-PROTAC to mediate degradation of target ABL and BCR-ABL proteins by means of UV light-controlled change in structural configuration (Figure 3) [33]. So, this promising light-operated photo-pharmacological technique provides a convenient approach for the optical control of intracellular protein levels and could lead to new types of precision therapeutics that avoid undesired toxic side effects [34].

Recently, an efficient two-stage strategy was developed for quick screening of multiple parameters for the development of potent PROTACs and applied for estrogen receptor alpha (ERα) degradation [35]. The first aldehyde–hydrazide coupling chemistry allowed generation of a library of sufficiently pure PROTACs with various E3 ligase ligands, linker lengths/types, linking positions that were appropriate for cell-based evaluation. After identification of an active degrader, the acylhydrazone moiety can be replaced by an amide group in the relatively flexible linker region to yield more stable and drug-like PROTAC with superior potency.

A new approach for the linker design was recently reported by Testa et al. [36], where a cyclizing PEG linker was used to form a macrocyclic PROTAC with increased target selectivity compared to the parent linear PROTAC. Despite a 12-fold loss in binding affinity for BRD4, macro-PROTAC exhibited cellular activity comparable to MZ1, one of the most potent PROTACs reported to date.

## Impact of the linker length

One of the most critical decisions in designing a potent PROTAC is choosing the right length of the linker connecting ligands binding POI and E3 ligase. The linker composition and length play a key role in potency and physicochemical properties of the PROTAC compounds. If the linker is too short, the two ligands cannot simultaneously bind to their respective proteins due to steric clashes resulting in failure of ternary complex formation. On the other hand, if the linker is too long then two proteins cannot be brought in close proximity with each other for target ubiquitination. Currently, structural optimization of PROTACs mainly focuses on the evaluation of structure-activity relationship (SAR) for various size linkers. Normally, the assessment begins with a longer version of the linker, and then the length is gradually decreased until the activity is lost.

The generalized study of PROTAC design by means of fine-tuning the linker length reported by Cyrus et al. [37], highlights the critical importance of linker properties in designing a potent ERα targeting PROTACs. Their results showed that while the 12- and 16-atom PEG linkers had similar binding affinities to ERα, the latter was significantly more potent in degrading this target. In case of homo-PROTACs (two same thalidomide moieties connected by PEG linkers of various lengths) for the degradation of CRBN the optimized linker was a short 8-atom long PEG [38]. PROTACs based on TANK-binding kinase 1 (TBK1) and VHL E3 ligases with linkers shorter than 12 atoms showed no apparent activity, unlike compounds with longer linkers that exhibited robust degradation potential [39]. In general PROTACs with longer linkers demonstrate higher efficiency for mediating degradation of the target proteins [40–44].

*In silico* methods also have been used to design new PROTACs and predict the structure of the formed ternary complex. This includes separately docking PROTAC compound into the appropriate ligand-specific pockets of POI and E3 ligase to estimate the minimal required linker length [45, 46]. However, despite the overall progress in computational methods the rational design of PROTACs still remains mostly empirical.

These studies, along with the others, highlight the need for new PROTACs to be tested with a range of linkers of various lengths. In addition, such parameters as linker thermodynamics and flexibility with regard to steric hindrances should be considered when designing a new PROTAC with potential therapeutic application [47, 48].

## Impact of the attachment site

Generally, PROTACs are optimized by determining a position for structure derivatisation that allows retaining maximal binding affinity. The linker attachment site also plays an important role in degradation of the target protein [43, 49]. The choice of the attachment site is typically guided by analysis of solvent-exposed areas at the protein-binding ligand interfaces. If a PROTAC linker has no capacity for further extension, then this limits the ability of an E3 ligase to mediate transfer of ubiquitin moieties to POI.

The early studies have shown that rational design and optimization of PROTACs for degradation of ERα can be expanded by attaching a penta-peptidic moiety at C7 and C16 positions of estradiol. The optimized linker connection at C7 position of estradiol generated a PROTAC with the highest affinity and degradation potential towards ERα [50]. Recently, researchers found that PROTACs with different attachment sites for VHL ligands yielded substantial target selectivity [51]. Based on the distinct linker attachment point and length, isoform-selective PROTACs for the p38 mitogen-activated protein kinase (MAPK) family differentially recruited VHL, resulting in degradation of p38α or p38δ.

**Figure 4.**
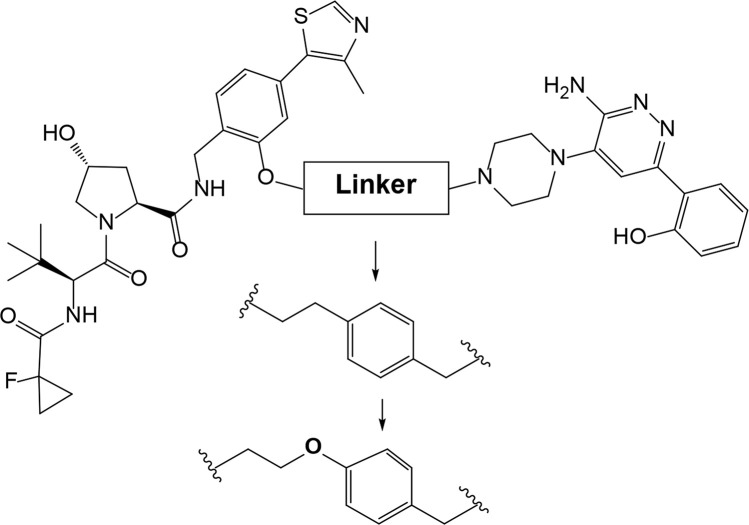
Rational structure-based design of BRD4 degrader PROTAC

Examining a repertoire of linkers with varying length and composition is essential for PROTAC development, especially given the modularity of synthetic approach. Careful design of the linker region may critically improve PROTAC affinity and selectivity. For example, in case of bromodomain and extra-terminal motif proteins (BET) domain protein BRD4 the successful PROTAC design has been achieved through the use of structure-based design [52]. Firstly, benzene component was incorporated to the linker, which allowed formation of π-π stacking interaction with Tyr98 of VHL. The optimized PROTAC was found to have better molecular recognition ability and improved stability of the ternary complex. Next, the researchers added an oxygen-atom to the linker to increase its length, achieve enhanced cell permeability and inducible degradation of a range of targets, e.g., SMARCA2, SMARCA4 and protein polybromo-1 (PBRM1) (Figure 4).

Although PROTACs represent a highly promising class of small molecules for drug discovery, still many concerns remain regarding their further development and clinical application. The primary aspects to be considered include cellular permeability and solubility, tissue distribution and metabolism–all of which can be improved by optimizing the PROTAC linker among other parameters. Another challenge is synthetic accessibility that implies optimization of the linker length and composition. Recent studies and discoveries in the PROTAC field demonstrate that linker is an essential component that modulates binding kinetics and substantially impacts the potency and selectivity. Thus, rational linker design including composition, length and attachment points represents one of the cornerstones in the development of potent PROTAC compounds.

## Conclusion

PROTAC-mediated ubiquitination and degradation of specific protein targets offers a previously inaccessible level of specificity and represents a promising strategy for post-translational regulation of intracellular protein levels. PROTACs demonstrate significant potential for innovative chemical intervention based on ubiquitin proteasome system and hold much promise as prospective drug candidates.

Challenges such as determination of optimal linker length and composition to achieve desired physicochemical properties are expected to be highly target-specific and, therefore, will need to be very carefully regulated on a case-by-case basis. The positive aspect is strengthened by the fact that many research groups around the globe began to successfully overcome these problems [53, 54]. Computer-aided design and novel smart strategies, such as PROTAC shortening, photoPROTACs, in-cell click-formed CLIPTACs, “click chemistry” for *in situ* synthesis provide access to expanded PROTAC libraries. In the future, creative and fruitful linker chemistry will open a window of opportunities for the discovery of new PROTACs for therapeutic application and treatment of various human diseases.

## Abbreviations

ABL: tyrosine-protein kinase
AR: androgen receptor
BCR: B-cell receptor
BD: bromodomain
BET: bromodomain and extra-terminal motif proteins
BRD: bromodomain-containing protein
CLIPTAC: click-formed proteolysis targeting chimera
CRABP: cellular retinoic acid-binding protein
CRBN: cereblon
EGFR: epidermal growth factor receptor
ERα: estrogen receptor alpha
PEG: polyethylene glycol
photoPROTACs: photo-switchable PROTACs
POI: protein of interest
PROTAC: proteolysis targeting chimera
VHL: Von Hippel-Lindau protein

## Declarations

### Copyright

© The Author(s) 2020.
